# Exploring community capacity: Karen refugee women’s mental health

**DOI:** 10.1108/IJHRH-02-2018-0025

**Published:** 2018-09-10

**Authors:** Nancy Clark

**Affiliations:** Faculty of Human and Social Development, University of Victoria, Victoria, Canada

**Keywords:** Community capacity, Mental health, Gender, Intersectionality, Structural violence, Cultural safety, Karen women, Postcolonial feminist

## Abstract

**Purpose:**

The purpose of this paper is to describe Karen refugee women’s experience of resettlement and the factors which structured community capacity to support their mental health and well-being.

**Design/methodology/approach:**

A postcolonial and feminist standpoint was used to bring Karen women’s voice to the knowledge production process. Data were collected through ethnographic field observation, in-depth semi-structured individual and focus group interviews with Karen women as well as healthcare and social service providers.

**Findings:**

Three interrelated themes emerged from the data: Karen women’s construction of mental health as “stress and worry”; gender, language and health literacy intersected, shaping Karen women’s access to health care and social resources; flexible partnerships between settlement agencies, primary care and public health promoted community capacity but were challenged by neoliberalism.

**Research limitations/implications:**

Karen women and families are a diverse group with a unique historical context. Not all the findings are applicable across refugee women.

**Practical implications:**

This paper highlights the social determinants of mental health for Karen women and community responses for mitigating psychological distress during resettlement.

**Social implications:**

Public health policy requires a contextualized understanding of refugee women’s mental health. Health promotion in resettlement must include culturally safe provision of health care to mitigate sources of psychological distress during resettlement.

**Originality/value:**

This research brings a postcolonial and feminist analysis to community capacity as a public health strategy.

## Introduction

The process of migration and the context of resettlement are determinants for the mental health and overall well-being of refugee women. Between 60 and 80 percent of the world’s refugees are women and girls (Raphael *et al.*, 2012). The percentage of refugee women at risk—that is, women who require resettlement based on their safety and well-being—has risen from 6.8 percent in 2007 to 11.15 percent in 2011 (United Nations High Commissioner for Refugees, 2013). Resettlement is recognized as a key protection tool for refugee women and girls at risk to ensure protection and well-being, but only if their gendered specific needs are recognized and addressed (United Nations High Commissioner for Refugees, 2013).

Refugee women’s mental health is impacted by resettlement factors related to complex gender role strain, lack of social support, low literacy and education, domestic violence and discrimination (Kirmayer *et al.*, 2011; Raphael *et al.*, 2012; Beiser, 2009). Basic literacy and health literacy are necessary for refugee women’s access to health care and settlement resources. For example, functional health literacy requires ability to utilize health resources, such as making medical appointments and navigating health care systems (Mitschke *et al.*, 2013; United Nations High Commissioner for Refugees, 2013; Williams and Meadows, 2017; Zanchetta *et al.*, 2012).

Research within western countries offering protection to refugees on humanitarian grounds consistently report that psychological well-being of refugee groups is related to complex post-migration factors which moderate the effects of pre-migration traumas and stress (Kirmayer *et al.*, 2011; Schweitzer *et al.*, 2011; Marlowe, 2010). Refugees are at higher risk for psychological distress including depression, post-traumatic stress disorder (PTSD), chronic pain and somatic health concerns with asylum seekers having ten times the rate of PTSD, and where exposure to torture is the highest predictor for PTSD (Kirmayer *et al.*, 2011). A study conducted with Burmese refugees in Australia found that the mental health status of refugees from Burma reported substantial psychological distress ranging from PTSD 9 percent; anxiety 20 percent; depression 36 percent and somatization 37 percent (Schweitzer *et al.*, 2011). The gendered effects of migration and post-migration are poorly understood, however, a Canadian systematic review found that immigrant women experience two to three times the risk of their Canadian born counterparts for post-partum depression (Kirmayer *et al.*, 2011).

Since 2005, Burmese ethnic minorities have been one of the largest resettled groups across Australia, Canada and the USA. Karen women and families represent one of the largest internally displaced Burmese ethnic groups across Southeast Asia (Citizenship Immigration Canada (CIC), 2011). These groups come from Mae La Oon and Mae Ra Ma Laung refugee camps and are considered the most difficult to reach, the most overcrowded and experience ongoing state-sponsored violence (CIC, 2011; Norrsworthy and Khuankaew, 2004). Studies with Karen (pronounced Kuh-REN) refugees resettled to the USA have shown that gender and education are important determinants of settlement and integration (Gilhooly and Lee, 2017; Hoffman and Robertson, 2016; Power and Pratt, 2012). A systematic review of Karen resettlement shows that mental health is related to a disruption of cultural collectiveness, and challenges to navigating the resettlement process, obtaining adequate housing and finding accessible education and health care (Sarah *et al.*, 2016). Gilhooly and Lee (2017) found that in some refugee camps Karen women and girls were excluded from education based on marital status, while Karen men and boys dropped school due to lack of English language proficiency and employment. Childhood education remains a main source of resettlement stress for Karen families (Gilhooly and Lee, 2017). Examining the contributions of gender and mental health outcomes, Schweitzer *et al.* (2011) found that gender was not associated with high levels of traumatization for ethnic Burmese, however, problems with communication, access to health and welfare services as well as worry about family overseas contributed significantly to post-migration stress.

Canadian immigration policies have instituted more liberal policies which offer protection for refugee groups through the Immigration and Refugee Protection Act (IRPA). However, multicultural societies such as Canada uphold a distributive justice framework, which value equality but do not consider the differential needs of immigrant women, of whom refugee women are a subset. This lack of recognition can perpetuate inequities and mask the human right to health placing the burden of responsibility for health on women (United Nations High Commissioner for Refugees, 2013). Health is a key human rights issue, and therefore lack of access to it (based on gender, race, age, class) is a form of systemic and structural violence (Farmer *et al.*, 2006; Galtung, 1969; Oxman-Martinez and Hanley, 2011; Quesada *et al.*, 2011). Despite recommendations made by the United Nations High Commissioner for Refugees (2013) on the resettlement of refugee women at risk, immigrant service organizations as well as primary care services lack adequate preparation for refugee women who arrive with historical violation of their human rights (Bartolomei *et al.*, 2014). Refugee identities, particularly those of women, reified by static categories where trauma is a master narrative as individuals requiring protection can negate the social and structural impacts on mental health (Aberman, 2014; Malkki, 1995; Marlowe, 2010; Schweitzer *et al.*, 2011).

## Theoretical approach

A critical ethnographic (CE) approach informed the analysis of social structures that shaped Karen women’s experiences of resettlement. Drawing on this approach, knowledge is generated for social justice aims (Carspecken, 1996; Madison, 2005; Thomas, 1993). Specifically, CE can uncover subtleties of oppression (Carspecken, 1996). Underpinned by social justice—postcolonial feminist theories, intersectionality and cultural safety—provide a lens to understand the factors that shaped Karen women’s mental health. Mental health is a construct that may have different cultural meanings among cultural groups. In this research mental health is defined broadly as “a state of well-being in which the individual realizes [their] own abilities, can cope with normal stresses of life, can work productively and fruitfully, and is able to make a contribution to [their] community” (World Health Organization, 2014, para 3). Multiple intersecting social, psychological and biological factors influence mental health, including poverty, low levels of education, rapid social change, gender discrimination, social exclusion, risk of violence and human rights violations (World Health Organization, 2014).

## Community capacity

Community capacity is defined as both a process and an outcome that requires collaborative action to promote healthy public policy and address systemic and structural inequities of community needs (Jung and Choi, 2013; Labonte and Laverack, 2001; Scott *et al.*, 2012). Postmodern critiques of community suggest that globalization and transnationalism challenge firmly held beliefs that a community is defined by local geographies (Pandey, 2005). Despite this challenge, existing sets of relationships where structural elements of social capital (network between governmental and non-governmental services) work to increase social support and community needs (McKenzie, 2008). Community capacity is the ability of local communities to mobilize existing resources to meet the evolving health needs of the community (Jung and Choi, 2013; Smith, 2008).

According to Jung and Choi (2013), conceptual understandings of community capacity include capacity building processes that are multidimensional, involve multiple community actors within social structures. The complex web of political, socioeconomic and historical processes implicates local community contexts in which resettlement occur (Smith, 2008). For example, in the wake of 9/11, newcomer women are particularly vulnerable to more subtle forms of oppression related to experiences of racial and linguistic discrimination (Piwowarczyk and Keane, 2007). Reforms underpinned by histories of colonialism, neoliberal values can fragment social capital, networks and relationships aimed at fostering community capacity.

Postcolonial feminist perspectives offer an analysis of the relationship between Karen women’s agency and existing structural policies and practices that were often embedded in historical “race” class and gender relations. Rooted in feminist discourse, intersectionality addresses simultaneous intersections between aspects of social difference and identity, i.e., race, class, gender and other categories of social difference such as language/literacy and education and the ways in these intersections sustain or reinforce inequities at macro and micro levels (Hankivsky *et al.*, 2010). Cultural safety is as a process and outcome of cultural competence (Kirmayer, 2012). Cultural safety used as an analytic lens can uncover how power relations are medicated between Karen women, health providers and broader social contexts. Combined, each of these perspectives has in common a shared concern for promoting social justice and human rights of marginalized groups.

## Purpose of the study

Refugee women continue to experience negative material life circumstances that do not end when resettled into nation states offering protection. This research foregrounds Karen women’s experiences and the social structural factors, which facilitated and/or challenged community capacity to support their mental health and well-being during resettlement in a western Canadian province. Data collection occurred over a 17-month period from May 2012 to end of September 2013, in a smaller suburban community in a western Canadian province.

## Method

### Recruitment and sample

Purposeful sampling in ethnographic research includes both the participants as a unit of analysis as well as a social context (Hammersley and Atkinson, 2007; Madison, 2005). Purposeful sampling occurred through recruitment posters and information letters distributed by community leaders working with an Early Years Refugee Project (EYRP), a non-profit service organization aimed to support early childhood education for refugee women with children between the ages of zero to six. After substantial analysis of the data was gathered, theoretical sampling was done to verify emerging codes and categories with Karen women (Coyne, 1997).

Individual and focus groups were conducted with Karen women (*n*=12) in S’gaw Karen language with Karen women who were key informants and leaders in their community. Karen women received child minding support and a small honorarium. Focus group interviews provided deeper understanding of issues identified within a group context (Krueger and Casey, 2009). In-depth individual and focus group interviews were held with community health and social service providers (*n*=26) who provided frontline health care and settlement services to Karen women. This sample included nurse practitioners, public health nurses, speech and language pathologists, registered dieticians, registered midwives, settlement workers, community volunteers, early childhood educators and managers.

The mean age of Karen women was 37 years (age range: 26–60 years); number of years in Canada ranged from 3 to 7 years; two women had obtained their Canadian citizenship; ten were permanent residents; all women identified as Christian; 11 women were born in Burma and 1 in Thailand; four women reported having schooling less than three years (33 percent); two women identified having up to eight years of schooling (17 percent); six women reported having at nine years of schooling (50 percent). The women’s highest level of education self-reported to be level 10, which may not be equivalent to Grade 10 in North American standards. Five of the women reported having attended English as a second language class during their resettlement. All the women (100 percent) were renting and living in lower-income housing. Ten Karen women (83 percent) reported that they were dependent on their spouse for income and received welfare and/or childcare subsidies, and two women were employed (one as a settlement worker the other had seasonal employment) (17 percent). The socio-demographic profiles of the women who participated are in Table I.

In total, 38 research participants were interviewed, 12 Karen women and 26 community health and social service providers. Table II provides a summary of the total participant sample. Ethical approval was received from the University of British Columbia, as well as the geographic health authority and non-government organization for this study. Informed consent from all research participants prior to interviews and observations was received.

### Data collection and analysis

A CE approach and women’s standpoint method of inquiry provided a rich account of Karen women’s perspectives on the factors, which supported and challenged their mental health. Standpoint method is a sociological method that begins with the actualities of women’s experience and aims to construct knowledge from women-centered perspectives (Smith, 1992, 2005). Understanding how things work in the everyday lives of Karen women included 1,632 hours of participant observations at an EYRP, attending medical appointments, home visits and community advisory meetings with Karen women and community service providers.

All interviews were transcribed verbatim, analyzed and stored using a qualitative software program (QRS N-Vivo 10^TM^). To ensure accuracy in language translation, an independent consultant analyzed discrepancies between the audio tape recordings and transcripts. Consulting included checking all the audio recordings conducted in S’gaw Karen language against the translated English transcripts. Women who provided language translation were also interviewed to make space for the inclusion of all women’s voices and increased the validity of the interpretation of the findings. Data analysis was iterative and included organizing themes into codes and categories (Creswell, 2013). The concept of positionality is central to CE approaches with social justice or emancipatory aims (Thomas, 1993; Madison, 2005). To attend to researcher positionality, the researcher used ethnographic field notes and Maxwell’s (1996) typology of validity to develop a reflexive critique on the data and representation of Karen women’s views. This included participant member checking to validate the findings. Standpoint method also positioned Karen women and the researcher, an immigrant woman, as part of a participatory knowledge production process.

## Results

Three dominant themes emerged from the data and include: Karen women’s construction of mental health as “stress and worry”; gender, language and health literacy intersected, shaping Karen women’s access to health care and social resources; community capacity was promoted through collaboration across settlement agencies, primary care and public health but challenged by neoliberalism.

## Resettlement and mental health—“a lot of stress and worry”

Although some Karen women arrived with histories of trauma, mental health was constructed as stress and worry related to children’s education, and access to health care. A young Karen mother explained her experience as stress:

P030: […] it is stressful to go and ask somebody to help you all the time […] And then, plus, if you don’t speak the language, you have to take your kids to the doctor and you need help. If you need to apply for renter assistance, you need help. Standing on other people’s feet is not the same as you walking by yourself.

In this context stress and worry is framed within a continuum of psychological distress and potential threat to Karen women’s mental health and well-being. Karen women faced increased psychological distress when faced with multiple barriers to accessing health and social services. “Walking by yourself” meant that women wanted to be independent but felt constrained without appropriate supports. At the same time, asking for help was constructed as “dependent other.” A community volunteer describes the impact of resettlement:

P0036: […] they all have […] housing rent assistance and every year they have to renew it. So this person has been here five years and every year someone fills it out for her. But we’re never teaching her how to fill it out right? So the last six or eight months I’ve really been trying to make people at least write their name, their address and their kids names and birth date like you have to be able to do that right? So really for the first number of years all frontline workers were guilty of it, we did everything for them. But yet they were just not in a mental space to be able to even try like they were just so overwhelmed with everything the basics of life […].

The resettlement needs of Karen families were high, given that most families had spent upwards of two decades in refugee camps where education is limited. A few Karen settlement workers provided Karen language services but could not provide outreach support due to lack of funding. Dependent “other” signifies the prevailing ethos of neoliberalism embedded in immigration policies that provide short-term funding contracts for resettlement agencies.

Lack of available interpreters was a major barrier to health care. A Karen woman who works as an interpreter noted:

P0011: she’s scared, she’s worried, she feels sorry to call the interpreter. Like for example, the last two days she called the interpreter and today she needs to go to see the doctor again. She worries. She waits and waits and waits until her daughter feels very sick […] she tries to close her eyes and call the interpreter. But when she sees the doctor, the doctor says “why you not come earlier? Your child is very sick. […] it will be too late.” But she says “I’m worried because I’m scared to call the interpreter”.

Health care is a fundamental human right and lack of access to it based on race, class, gender, culture and other social differences are forms of systemic violence because they cause harm to people (Galtung, 1969; Farmer *et al.*, 2006). Women were reluctant to call upon members of their own ethnic community who provided translation. In particular, Karen women with lower literacy and education were dependent on other Karen women with higher education. This created cultural vulnerability and stigma. Cultural safety was described by a health provider:

P0044: […] patients will refuse to use certain interpreters because they just don’t feel comfortable or they don’t feel safe.

Women with higher education and English language skills often took on the role of interpreters within their community. Karen women often called those perceived as highly educated as theramu (for a woman) or thera (for a man) which means “teacher.” This cultural norm also had implications for construction of Karen women as dependent other. Simultaneously, settlement workers who were trusted and perceived to have higher status because of their education and class position, were viewed by Karen families as potentially facilitating better access to health services. Asking women, who they trusted as interpreters, spending more time during medical appointments and knowing something about their history was perceived as culturally safe by Karen women.

A gendered responsibility to provide health care and education for their children compounded women’s stress. All the Karen women who participated in this study mentioned that their children’s health and access to good education were primary reasons for coming to Canada. A young Karen mother further describes these gendered experiences:

P0030: A lot of stress. For women just have to deal with the children and have to […] yeah, after looking after the children and then they – some of them feel lost, like my children don’t listen to me because I don’t speak English. I don’t know how to raise my kids in this country.

A postcolonial feminist analysis suggests that migration and resettlement of the Karen does not necessarily provide opportunities for education given women’s gendered responsibilities for family health, as many women negotiated and re negotiated their agency in the context of accessing health care.

## Intersections of gender, language and health literacy—“it’s invisible […]”

Gender as a determinant of mental health was invisible. Some service providers assumed that being able to speak English was equivalent to being health literate. In the following excerpt, a Karen woman explains:

P025: […] my English is not good […] I have a big problem when I first came […] my daughter she’s very sick, [name] she has a high fever […] but how can I go to see the doctor, I ask my counsellor. She said I’m very busy now I can’t take you to see the doctor and then she said can you speak English? I said I can speak just a little bit […]. she give me the map you can go to see the doctor, you can go there […] I feel very upset the whole night I can’t sleep […] and then I ask my counsellor again she said I’m sorry you can speak English so you can go by yourself […] why I come to Canada like this?

For many women, their gendered responsibilities of childcare, their lack of language resources, and lower literacy were impacted by structural reforms. Being able to navigate and access health care resources independently required not only language ability but also functional aspects of health literacy such as being able to read street signs and/or system navigation, and how to make medical appointments.

There was a general perception that Karen women are more educated. A male Karen settlement worker explains this paradox:

POO8: Well like because like she’s [more] education than the husband right? And she know[s] how to get around […] It’s not common, […] but in the Karen community I believe the women are learning more than the men […] see the difference between the husband and wife when I work with them […] some people will say that Karen, woman need more help than the man whatever you said it. But sometimes it depends on family […].

Gender roles, identities and gender relations structured the kinds of services and supports offered. Karen women were learning to navigate health services and supports as well as gain access to literacy education through EYRP’s. A program manager described this advantage:

POO41: And so I think the mums have got an advantage somewhat over the dads because the dads were […] hearing we don’t see again it’s kind of somewhat invisible […].

Gender was invisible in the context of reforms to immigrant service agencies such as EYRP’s where women only programs structure domestic work, labor and education. Domestic work was predominantly the role of young Karen mothers; however, older grandparents and fathers also provided child rearing. This finding underscore gender specific influences on inequitable settlement support.

## Community capacity—“you have to work from a different model”

Community capacity, a health promotion strategy used to identify community health needs requires a multilevel approach (Jung and Choi, 2013). Community collaboration between frontline—volunteers, community stakeholders and health service providers—promoted mental health of Karen families. However, lack of knowledge about the Karen put increased responsibility on local volunteers to advocate for more resources. A community support worker highlights the implications of lack of community preparedness:

P019: […] I’ve had it with someone who was almost at the point where she could go to simple appointments by herself […] I was encouraging her go up to the desk, show your care card and they’re going to ask your name and your address and I’ll just stand back and you just try it. […] so the lady asked for her card and then what’s your address? […] she said her apartment number and her building number backwards, […] so then I stepped in and said it’s actually apartment [number], the building number is this. The receptionist grabbed the care card, threw it on the desk and said let me talk to someone who speaks English I don’t have time for this!. So that just set that girl back like for a year […] she was too afraid to go to a doctor by herself right?

Lack of community preparedness excluded women and families from accessing health services. For Karen women, knowing about their pre-migration context was important for promoting culturally safe health care services and mitigating systemic forms of exclusion. Health promoting strategies also required community collaboration across services. A frontline service provider explains:

PO36: It will be important that […] those, agencies are talking […] it would be a shame for that to stop so hopefully that will keep going […] Without that, I think [City B] would be a lot more fragmented and […] people would fall through the cracks a lot more. But because we get together regularly […] [to] make sure that no one is falling through the cracks. If certain programs are doing certain things, then we can do something different. […] And start collaborating as a community.

Collaboration among different agencies was as a strategy to prevent silos: a process in which services operate in isolation from each other. A public health nurse advocated for “[…] a different model” for public health, which integrates diverse health needs of refugee groups and enhanced cultural knowledge.

Community capacity building perceived as a process, in flux-required adaptation and flexible policies to adapt to diverse needs of Karen women and families. The act of collaborating assisted various community stakeholders, public partners and service providers to draw on community strengths. As a byproduct, community capacity building was a form of resistance to redress the fragmentation of services brought about neoliberalism.

## Limitations

Language is inseparable from the values of those conducting translation and/or interpretation (Creese *et al.*, 2011; Temple and Young, 2004). This is salient in conducting research with Karen women who arrived in groups where they have historical relationships with camp members. To increase validity of findings, Karen women with higher language skills provided translation and also facilitated consent. Karen women who were comfortable sharing their stories with women they trusted, provided translation. Karen women represent a unique cultural group with distinct histories of colonization and therefore findings may not be applicable to other refugee women.

## Discussion

### Resettlement as a gendered experience

This research brings attention to Karen women’s standpoint to explore how colonial relations in the context of refugee resettlement shape systemic processes of health inequity. In the sociopolitical context of settlement reforms, an ethos of neoliberalism reinforced vulnerability and dependency affecting Karen women’s identities. The intersections of gender, health literacy and education created simultaneous privilege and disadvantage for Karen women.

This finding is consistent with feminist thinkers who advocate for a power analysis, which situates women’s health, constructed by patriarchal values, in the context of broader social determinants of health (Ponic *et al.*, 2014; Racine and Lu, 2015; Zanchetta *et al.*, 2012). Gilhooly and Lee (2017) research showed that gender roles for Karen women and girls tend not to change during resettlement. This structural challenge can be redressed through resettlement policies, which include participation, recognition and redistribution of resources. The work of feminist political scientist and philosopher Nancy Fraser (1999) argues for a normative paradigm in which cultural domination and injustice caused by economic redistribution integrates concurrently with a politics of recognition and parity of participation. This requires a recognition of how refugee women and men’s work distributed by “labour” work and “unpaid” or domestic work. Importantly refugee groups must have a voice in policies and practices that directly affect their health. Post-migration mental health for women, who arrive in western countries under at risk visas and who require resettlement protection on human rights grounds, is linked to economic stability and employment (Kirmayer *et al.*, 2011).

Moreover, the politics of recognition examines cultural and social patterns, which reinforce oppressive structures, and exploits gender-defined roles. The findings of this research are consistent with Tastsoglou *et al.* (2014), who suggest, “Canadian immigration and refugee policies devalue women, create dependency and promote gendered power imbalances” (p. 69). Community capacity requires immigration policies to integrate the differential education, gender, and health literacy needs of refugees to provide equitable health care and build healthy public policy. This would include critical reflection on the structural processes of inclusion and exclusion.

### Promoting culturally safe community capacity

The UNHCR considers the Karen, and other ethnic minority groups, e.g., Rohingya in Burma, in need of third-country resettlement because of ongoing state-sponsored violence and decades of political and cultural oppression (South and Jolliffe, 2015). The United Nations High Commissioner for Refugees (2010) defines resettlement as a process, which provides “protection to refugees when their lives, liberty, safety, health or other fundamental human rights are at risk in their country of asylum” (p. 18). Canada is one of 147 nations to have signed the UN Convention on Refugees and offered protection to Karen refugees.

The Canadian implementation of IRPA represents a landmark in Canadian immigration policy because it has removed barriers that previously limited the admission of immigrants based on their education, chronic disease and social capital. Between 2006 and 2009 approximately 786 Karen government assisted refugees were offered protection, through a group settlement initiative but continue to experience psychological distress due to limited support and lack of accessible health care services (Marchbank *et al.*, 2014). Lack of educational opportunities, inadequate translation services, worries about family left behind and employment are cited as major barriers to integration across international reviews on diverse ethnic Burmese (Gilhooly and Lee, 2017; Hoffman and Robertson, 2016; Schweitzer *et al.*, 2011).

The United Nations High Commissioner for Refugees (2013) has recommended that there must be culturally appropriate services for refugee women in places of resettlement, to redistribute resources, i.e., differential support for English language and literacy training to support women’s health. Redistribution of resources requires increased political advocacy to build pathways toward equitable health care services. Mental health policy and practice must attend to the day-to-day stress and worries refugee women experience to support their integration and overall well-being.

Cultural constructions of mental health narrowly defined by pre-migration trauma may overshadow refugee women’s day-to-day experiences of stress and the social etiology of mental health, thereby abdicating social responsibility for health on refugee women and families. Moreover, mainstream responses to understanding trauma as part of refugee experiences focus on a single traumatic event and reinforce passivity and dependency, which masks the social and ordinary lived experiences of mental health (Marlowe, 2010). Adopting cultural safety can promote community capacity through systemic reflexivity of historical colonial relations, which reproduce inequalities over time. Policy and practices can begin to shift the gaze away from discourses which conflate refugee women’s identity as dependent, toward practices that support women’s agency. As Smith (2008) notes community adaptation is a two-way process that requires behavioral flexibility of community structures an orientation toward what refugees have to offer, i.e., cultural capital.

Subtle forms of oppression can potentially re-traumatize and cause harm, through systemic policies that do not recognize and integrate refugee women and their gendered, education and literacy needs. Intersections of cultural, ethnic and gender ideals may compound levels of stress. These factors impacted Karen women’s mental health and are necessary for gaining access to employment, housing, social support and health care, which are main factors for successful integration and well-being (Gilhooly and Lee, 2017; Hoffman and Robertson, 2016; Power and Pratt, 2012; Racine and Lu, 2015). Restructuring the environment of settlement support and public health is necessary to uphold nation states such as Canada’s commitment to human rights and international convention of the UNHCR, which aims to protect the rights of refugee women.

### Recommendations for building healthy public policy

Development of community advisory groups with refugee women can strengthen community action and service planning. Community capacity to support Karen women’s mental health and well-being during resettlement in the Canadian context used capacity building strategies fostering advocacy, and flexible partnerships between settlement agencies, and public health. Aspects of community capacity building (collaboration and partnership) can build social capital and social networks (Williams and Meadows, 2017). Evaluation of community capacity initiatives must include gender, literacy and education as key factors for integration. This could include policy which facilitates use of interpreters across health systems. Patient and or peer navigators (PNs) can be used to promote culturally safe accessible health system navigation. PNs are used with many underserved groups to decrease barriers to health care (Chin *et al.*, 2012; Koh *et al.*, 2010). This initiative may be a useful approach to developing outreach support for refugee women and families with lower levels of health literacy.

Promotion of welcoming environments in primary care starts with gatekeepers. Leanza *et al.* (2014) advise that gatekeepers play a crucial role in setting the tone of the agency. Gatekeepers can ask simple questions such as “Are you new in Canada?” and “Would you like to have an interpreter?” and that the training of all staff, including reception staff, should be prepared to facilitate consultation appointments with newcomer women. Health system flexibility, particularly for altering appointment systems could include rescheduling services on a drop-in basis, which would also address the complexity of making medical appointments and exclusionary health care practices, which tend to locate the problem within the individual rather than as a feature of the organizational practice.

## Conclusion

While there is a call to provide protection for refugees across western nation states, international resettlement policy for refugee women and girls must attend to community evaluation of the intersecting dimensions which impact women’s health (United Nations High Commissioner for Refugees, 2013). Community capacity evaluations must include community contexts and the multiple, axes of social difference (education, migration history and gender) which construct Karen women’s identity. National immigration policy must guard against neoliberal politics shaping inequitable health resources which impact local community capacity to support integration of refugees. Overall, this study suggests that community capacity to support the mental health and well-being of Karen women must include a reorientation to the social determinants of mental health and greater flexibility across health and institutional systems to promote community capacity. Karen women’s standpoint offers a view to inform community capacity structures, which address intersectional dimensions affecting their mental health and well-being.

## Figures and Tables

**Table I:** Karen women’s demographic data

*Age*	
Mean	36.7
Min	26
Max	60

*Citizenship*	
Canadian permanent resident	2 (17%)
Citizen	10 (83%)

*Religion*	
Christian	12 (100%)

*People in household*	
Mean	4.9
Min	3
Max	8

*Number of children*	
Mean	2.5
Min	0
Max	6

*Education*	
Level 3 or lower	4 (33%)
Level 4–8	2 (17%)
Level 9 or higher	6 (50%)

*ESL classes*	
Yes	5 (42%)
No	7 (58%)

*Housing*	
Rental	12 (100%)

*Source of income*	
Dependent of spouse/child subsidy	10 (83%)
Employed	2 (17%)

*Years in Canada*	
Mean	5
Min	3
Max	7

**Note:** *n*=12

**Table II:** Total participants

Participants	Number of focus groups	Number of focus groups participants	Individual interviews	Total participants
Karen women	2	8	4	12
Health care providers	1	7	4	11
Settlement workers, community support workers and volunteers	1	6	9	15
Total	4	21	17	38
